# Electronic Band Structure of Gallium Sulfide (GaS) with Thickness Reduction Unveiling Parabolic and Pudding Mold Band Dispersion

**DOI:** 10.1021/acs.jpcc.4c08588

**Published:** 2025-02-05

**Authors:** Ashraf Abdelrahman Assadig Elameen, Debasis Dutta, Songül Duman, Marcin Rosmus, Gianluca D’Olimpio, Bekir Gürbulak, Danil W. Boukhvalov, Amit Agarwal, Antonio Politano

**Affiliations:** † Department of Physical and Chemical Sciences, University of L’Aquila, via Vetoio, 67100 L’Aquila (AQ), Italy; ‡ Department of Applied Science and Technology, Polytechnic University of Turin, Corso Castelfidardo, 39, 10129 Turin, Italy; § Department of Physics, Indian Institute of Technology Kanpur, Kanpur 208016, India; ∥ Basic Sciences Department, Faculty of Sciences, Erzurum Technical University, Erzurum 25050, Türkiye; ⊥ National Synchrotron Radiation Center SOLARIS, Jagiellonian University, Czerwone Maki 98, PL-30392 Krakow, Poland; # Department of Physics, Faculty of Sciences, Atatürk University, 25240 Erzurum, Türkiye; ∇ College of Science, Institute of Materials Physics and Chemistry, Nanjing Forestry University, Nanjing 210037, P. R. China

## Abstract

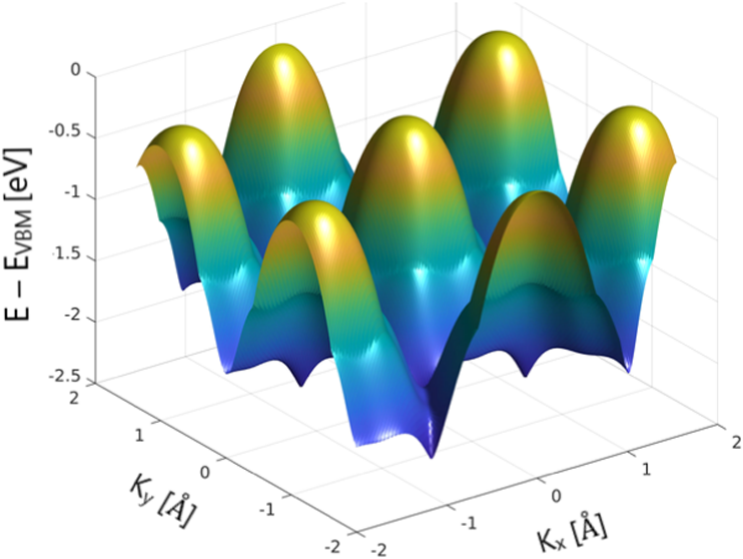

Metal monochalcogenides (MXs) have attracted significant interest due to their unique electronic properties, which can be tuned by varying the thickness. Gallium sulfide (GaS) stands out among MX compounds for its potential in photocatalysis, thanks to its bandgap within the visible range. However, the theoretical predictions of its band structure have not been experimentally validated until now. To bridge this gap, we performed angle-resolved photoemission spectroscopy (ARPES) measurements on bulk GaS to investigate its electronic band structure which revealed that the VBM is located at the Γ point, and from the analysis of isoenergy contours just below the Fermi level, the contours are relatively circular and centered around the Γ point indicating a high degree of isotropy and symmetry in the electronic states. Additionally, density functional theory (DFT) calculations revealed that the valence bands are composed of Ga 4s, Ga 4p, and S 3p orbitals, while the deeper bands are from S 3s orbitals. Furthermore, the theoretical calculations are extended to monolayer, two-layer, and three layer to observe the evolution in the band structure. Our results highlight a unique “Pudding Mold” valence band maximum (VBM) at the Γ point, featuring multiple maxima dispersed throughout the Brillouin zone. When the GaS sample is thinned to monolayers, this band transforms into a “Pudding Mold” shape, characterized by significant corrugation at the Γ point. This transformation predicts an increase density of states (DOS), which is highly advantageous for photocatalysis. The higher DOS enhances the absorption and utilization of visible light, which is essential in photocatalytic applications, and also provides more active sites for catalytic reactions.

## Introduction

The presence of a bandgap in layered van der Waals (vdW) semiconductors^1−5^ enables their use in optoelectronic devices,^6,7^ field-effect transistors,^8−10^ gas sensing,^11^ and photocatalysis.^12,13^ Bandgap engineering in vdW semiconductors is commonly used to optimize a specific property that can boost the performance of targeted applications.^14,15^ Among vdW semiconductors, group III–VI metal chalcogenides (MX), where M is Ga or In, and X is a chalcogen atom (S, Se, Te),^6,16,17^ provide several advantages, including superior tunability of the bandgap, high optical absorption in the visible range of the electromagnetic spectrum, and high carrier mobility.^18^ While the majority of III–VI MX compounds have indirect bandgaps in their bulk form and a direct bandgap in the monolayer regime,^19−21^ GaS uniquely exhibits an indirect bandgap in both the bulk and monolayer.^15,22^ The persistence of an indirect bandgap in GaS, even at the monolayer thickness, could lead to specific benefits. For instance, indirect bandgap materials are known for their prolonged carrier lifetimes compared with their direct-gap counterparts. This is because the momentum conservation rule requires the involvement of phonons to facilitate electron transitions between the valence and conduction bands, which typically results in lower recombination rates.^23,24^ Consequently, devices that rely on sustained carrier dynamics, such as photodetectors and solar cells, could benefit from such properties because they rely on longer carrier lifetimes to enhance the device performance.^25^ Moreover, the relatively small energy difference between the indirect and nearest direct bandgaps in GaS suggests that, with a modest input of thermal energy, carriers can be excited to the direct bandgap, potentially leading to pseudodirect transitions under certain conditions. This hybrid behavior of GaS can be exploited to tailor material properties through external stimuli, such as light and temperature, allowing for tunable electronic and optical characteristics. These properties are particularly useful in photoconversion processes. Recently, GaS nanosheets have shown remarkable performance in photoelectrochemical devices,^26^ with maximum responsivity of 6.8 mA W^1–^ in 1 M KOH at 1.1 V versus reversible hydrogen electrode (RHE) under 275 nm illumination with an intensity of 1.3 mW cm^–2^.

Despite the availability of theoretical predictions on the band structure of bulk GaS,^21,27,28^ but there is no experimental validations by ARPES experiments, which are crucial for direct observation of the electronic band structure. The shape of the valence band plays a crucial role in determining the mobility of charge carriers, which directly impacts the efficiency of various applications, including photocatalysis. In particular, the curvature and dispersion characteristics of the valence band influence the transport and mobility of charge carriers.^29^ Thus, a well-designed valence band structure can enhance carrier mobility.^30^ In photocatalysis, higher carrier mobility facilitates more efficient separation and migration of photogenerated charge carriers, which are essential for driving the catalytic reactions.^31^ Therefore, understanding and optimizing the shape of the valence band is vital for advancing the functionality of GaS in these applications, also in consideration of the promising implementation of photoelectrochemical devices.^26^

Here, we demonstrated by synchrotron-based ARPES measurements that the bulk GaS exhibits parabolic dispersion symmetrically dispersed around Γ point but this dispersion is transformed, as predicted by DFT calculations, into “Pudding Mold” shape for 1L, 2L, and 3L, which includes a parabolic feature that enhances carrier mobility and a flat region that increases the DOS, which are crucial for applications such as photocatalysis. Previously, the “Pudding Mold” shape of the valence band, observed in Na_*x*_CoO_2_^32^ was shown to enhance thermal power and improve conductivity while simultaneously increasing the power factor.^32^ Similarly, in GaS, the “Pudding Mold” shape is expected to enhance carrier mobility and increase the DOS. By understanding and optimizing the electronic band structure of GaS, one can enhance its performance in photocatalytic applications, making GaS a promising material for energy conversion and environmental remediation.

## Methods

### Single-Crystal Growth

The Bridgman-Stockbarger method was used to grow the single-crystal GaS ingots. Initially, a cleaned quartz glass ampule with a length of 250 mm and an inner diameter of 10 mm was filled with a stoichiometric amount of high-purity Ga and S, corresponding to 20 g of the final compound. A diffusion pump was used to evacuate it to a pressure of approximately 10^–6^ Torr, and an oxygen-acetylene torch was used to close the ampule. The ampule was placed in a handmade furnace in two different temperature zones, and the growth process was performed according to the temperature program.

### XRD

To determine the crystal structure of bulk GaS, XRD measurements were conducted using a Bruker D2 Phaser diffractometer, which utilizes Cu–Kα radiation (1.5406 Å) in the scanning mode from 5°to 90° 2θ with a step size of 0.02°.

### Theoretical Calculations

We performed first-principles calculations using the Vienna ab initio simulation package (VASP)^33^ within the Perdew–Burke–Ernzerhof generalized gradient approximation (GGA).^34^ We performed first-principles calculations using the Vienna ab initio simulation package (VASP)^33^ within the Perdew–Burke–Ernzerhof generalized gradient approximation (GGA).^34^ We used a plane-wave basis set with a cutoff energy of 450 eV. Spin–orbit coupling was considered using the second variant method. The electronic charge density was calculated using a γ-centered *k*-grid of dimensions 12 × 12 × 4. Electronic optimizations were performed with a tolerance of 10^–7^ eV, and a Gaussian smearing of 0.04 eV was used to compute the total energy. The projected orbital band structure was calculated using VASP. The van der Waals interactions between the layers were considered using the DFT-D3 method.^35^ To construct an effective tight-binding model, we employed the VASP2WANNIER90 interface.^36^ We constructed Wannier functions based on the dominant orbital contributions near the Fermi level using Ga s and p orbitals and S p orbitals. The surface spectral function was calculated using the recursive Green’s function approach in a semi-infinite geometry, as implemented in the WannierTools package.^37^

### ARPES

High-resolution angle-resolved photoemission studies were conducted at the URANOS beamline of the Solaris Synchrotron^38^ in Kraków, Poland.^38^ in Kraków, Poland. The beamline was equipped with a Scienta-Omicron DA30-L electron analyzer. The samples were mechanically exfoliated using adhesive tape under ultrahigh vacuum (10^–8^ mbar) at room temperature. The pressure during the ARPES measurement was maintained below 5 × 10^–11^ mbar and the temperature during the experiment was maintained at 79 K. During the ARPES measurements, significant charging of the samples was observed owing to incident light. Based on our observations, which indicate that visible light induces a shift in the electronic structure toward higher kinetic energies, we experimentally determined that illumination with a yellow LED is the most effective method to counteract this charging effect and stabilize the system during measurements. Thanks to the use of this light source, ARPES measurements could be conducted for many hours without any visible shift in the electronic structure.

## Results and Discussion

**Figure 1:**
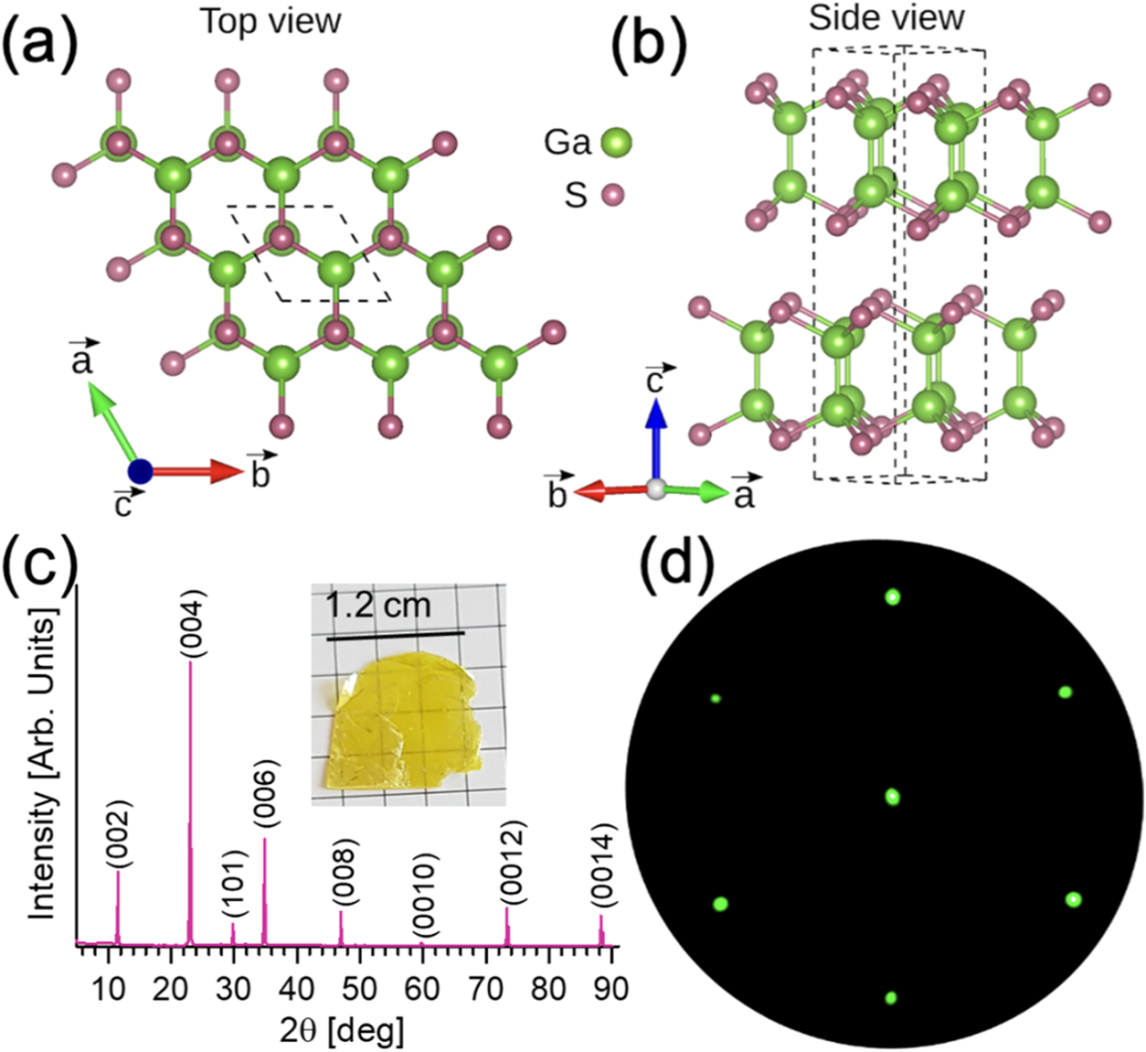
(a) Top and (b) side views of the atomic structure of GaS, Green and red balls denote Ga and S atoms, respectively. (c) X-ray diffraction (XRD) pattern of grown single-crystal GaS, whose photograph is shown in the inset. (d) low energy electron diffraction (LEED) pattern.

GaS is stacked along the *c*-axis through weak van der Waals forces, whereas in-plane atoms are held together through covalent bonds.^39,40^ Among the several possible polytypes,^15,41^ gallium sulfide crystallizes from the melt as hexagonal β-GaS; space group no. 19 (*P*6_3_/*mmc*,^42−44^Figure 1a,b).In β-GaS, the Ga and S atoms in each layer align directly with Ga and S atoms in the neighboring layer.^45^ The unit cell of the hexagonal GaS phase is composed of two S–Ga–Ga-S quadruple layers,^42,46^ with prevailing covalent intralayer bonding and negligible ionic contribution,^47,48^ whereas weak interlayer van der Waals forces regulate the interlayer cohesion along the optical *c*-axis.^8,49^ The β-GaS phase is particularly stable and can undergo a reversible polymorphic transition to ε-GaS only at applied pressures exceeding 3 GPa. The primitive unit cell of the hexagonal β phase has a lattice parameter *a* = 3.596 Å and *c* = 15.550 Å, as confirmed by X-ray diffraction (XRD) (Figure 1c), in agreement with previous investigations.^50−53^ The low-energy electron diffraction (LEED) pattern of the as-cleaved surfaces revealed sharp, intense spots, forming a distinct hexagonal pattern, which is indicative of surface crystallinity (Figure 1d).

To model the electronic properties of bulk GaS, we performed ab initio calculations using DFT with projector-augmented wave pseudopotentials, as implemented in the Vienna ab initio simulation package (VASP). The exchange and correlation potentials were treated using the Perdew–Burke–Ernzerhof (PBE) implementation of the generalized gradient approximation (GGA) (see Methods).

**Figure 2:**
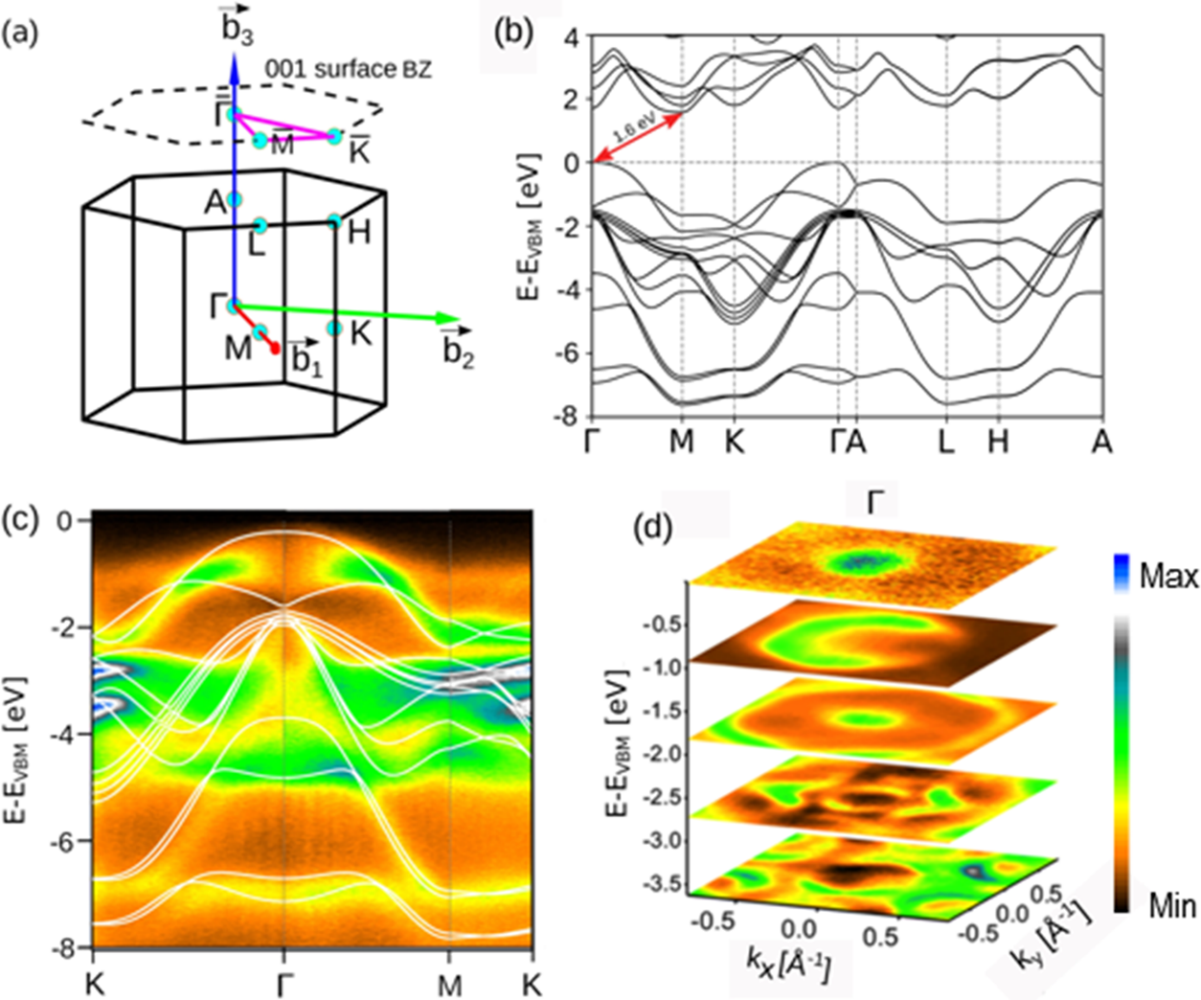
(a) Hexagonal bulk Brillouin zone (BZ) of GaS with different high-symmetry points. The upper dashed hexagon represents the BZ of the (001) surface (b) Electronic band structure of bulk GaS with spin–orbit coupling (SOC) included. The indirect bandgap estimated by DFT calculations is 1.6 eV. (c) Experimental ARPES spectrum of the as-cleaved surface of bulk GaS. The theoretical electronic structure was superimposed on it. (d) isoenergy contours around the center of the Brillouin zone.

The theoretical electronic band structure of bulk β-GaS is shown in Figure 2b. Bulk GaS maintains both inversion and time-reversal symmetry, causing all bands to be doubly degenerate. The calculated electronic band structure, including spin–orbit coupling (SOC), shows that the VBM and conduction band minimum (CBM) of bulk GaS are located at Γ and M points, respectively, resulting in an indirect bandgap of 1.6 eV (see arrow in Figure 2b). This result is consistent with previous theoretical predictions.^28,45^ However, it is worth pointing out that the functional employed in DFT calculations is known to systematically underestimate bandgap values.^54^

**Figure 3:**
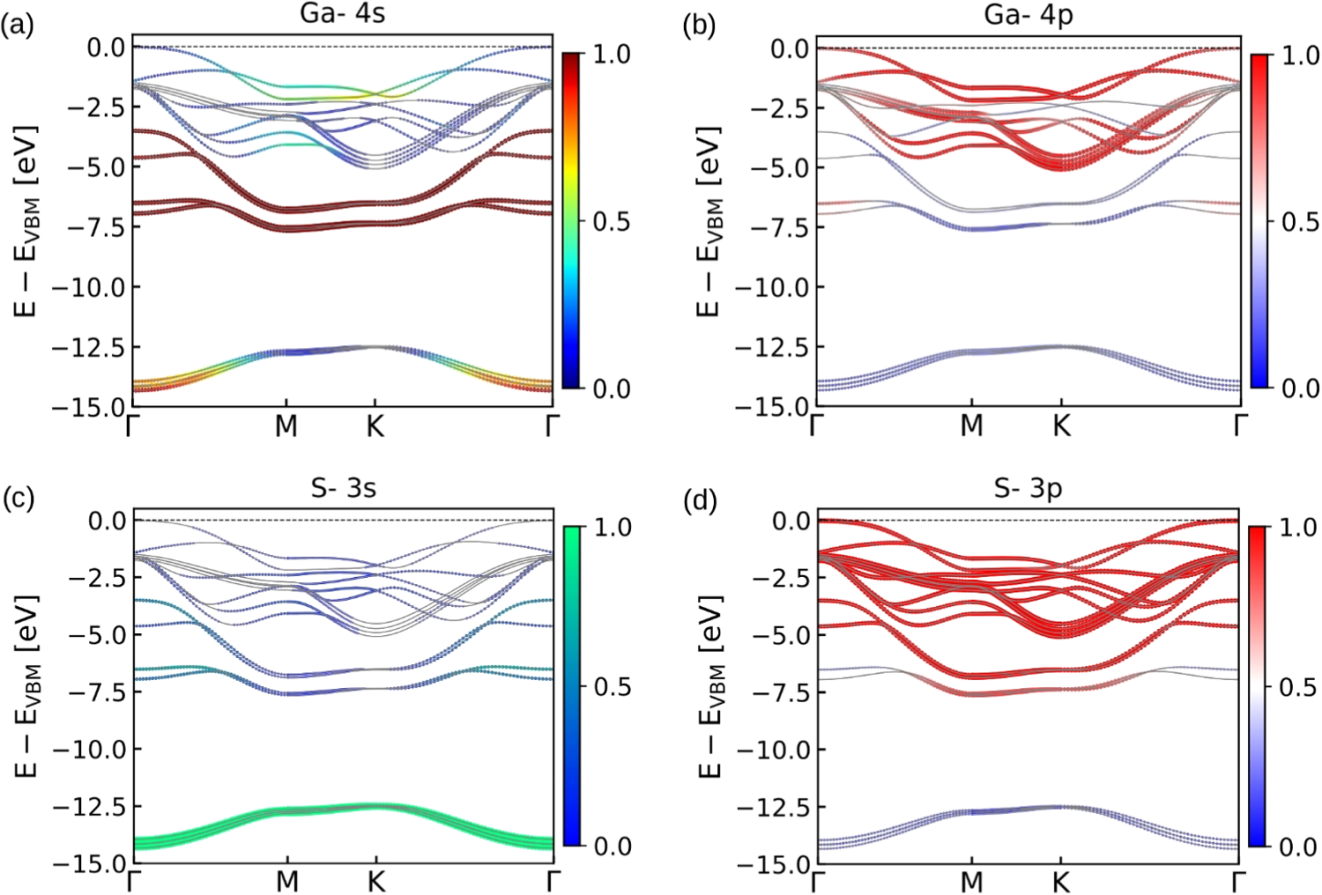
Orbital projected band structure of bulk GaS with SOC for (a) Ga-4s, (b) Ga-4p, (c) S-3s, and (d) S-3p atomic orbitals. The color map represents the orbital weight factor on various bands, having a maximum value of 1.

In Figure 2c, the ARPES spectrum of the as-cleaved surface of bulk GaS, overlaid with the theoretical electronic structure, reveals significant details about the electronic bands. The measurements were conducted with a photon energy of 100 eV and horizontal linear polarization. The valence bands are primarily composed of contributions from Ga 4s, Ga 4p, and S 3p orbitals (see also Figure 3), while the deeper bands are from S 3s orbitals. The parabolic nature of the band near the Γ point suggests low effective mass for charge carriers, which is beneficial for mobility. In the Γ–M direction, the bands flatten out, indicating an increase in the DOS.

Figure 2d depicts isoenergy contours around the center of the Brillouin zone, specifically focusing on the Γ point. These isoenergy contours provide a detailed map of the energy distribution in the *k*_*x*_–*k*_*y*_ plane at various binding energies. Each contour plot represents a constant energy slice through the electronic band structure, illustrating how the energy levels vary with momentum. At an energy level just below the VBM, the contours are relatively circular and centered around the Γ point, indicating a high degree of isotropy and symmetry in the electronic states. At lower energy levels, the contours begin to show more complex structures, revealing the anisotropic nature of the electronic dispersion in these regions. This complexity indicates variations in the effective mass of charge carriers and the potential for anisotropic charge transport properties. The smooth and continuous nature of these contours, particularly near the VBM, suggests a relatively flat energy landscape. This flatness corresponds to a high DOS in these regions, which is beneficial for applications requiring enhanced DOS, such as photocatalysis. The high DOS implies more available electronic states for catalytic processes, thereby facilitating more efficient charge separation and transfer during photocatalytic reactions.

Combining this with the orbital-projected band structure shown in Figure 3, it is evident that the contributions of Ga 4s orbitals are primarily observed between −3 and −8 eV, with significant presence at energies deeper than −14 eV. Around the Γ point, these bands show parabolic dispersion, indicating their role in carrier mobility. The Ga 4p orbitals have a strong presence near the VBM at the Γ point and, along the whole Brillouin zone, they span from −2 to −5 eV, also contributing significantly to the conduction bands starting above the Fermi level. The S 3s orbitals contribute to the deeper bands, primarily found below −11 eV, with little impact near the VBM. The S 3p orbitals dominate the valence band region between −2 and −7 eV, significantly influencing the VBM, especially at the Γ point, contributing to the flat band structure observed, which impacts the DOS.

**Figure 4:**
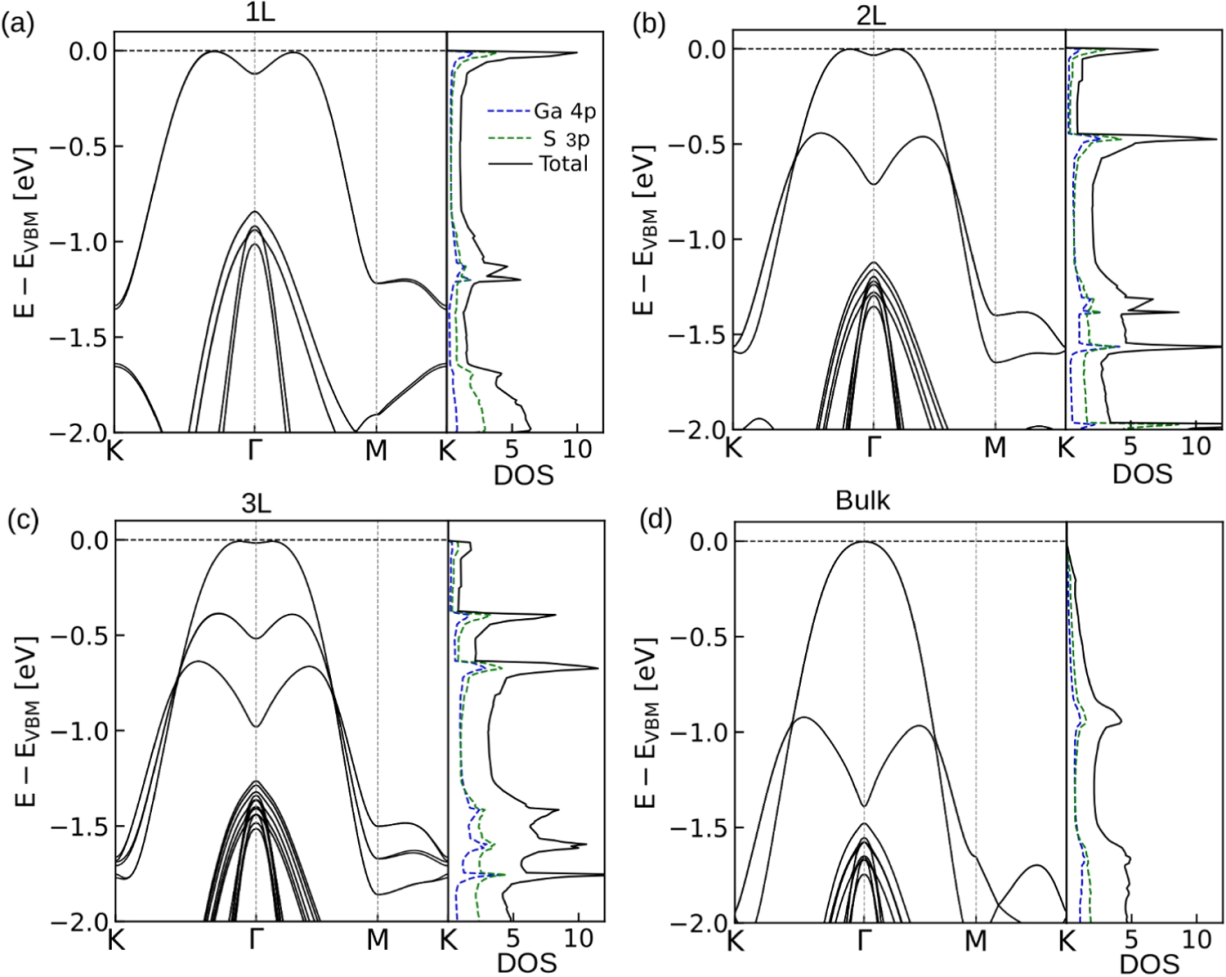
Band dispersion and DOS (1/unit cell·eV) of (a) single-layer (1L), (b) bilayer (2L), (c) trilayer (3L) and (d) bulk GaS in the presence of spin–orbit coupling. The first valence band of single-layer GaS shows a Mexican-hat-like energy landscape with a minimum around Γ point. This unusual band dispersion leads to a van Hove singularity in the DOS spectrum of the VBM. Interestingly, with increasing number of layers, the Mexican hat-type dispersion transforms into a parabolic band with a flat region at the topmost part.

In Figure 4, the evolution of the band dispersion from single-layer (1L) to bulk GaS is depicted, illustrating how the electronic structure changes with varying thickness from bulk to monolayer.

For the single-layer GaS, as shown in Figure 4a, the valence band exhibits a distinctive “Mexican hat” energy surface around the Γ point, characterized by a local minimum that creates a high DOS and a van Hove singularity (VHS) near the VBM. This feature near the Fermi level can induce a Stoner-type magnetic instability upon hole doping, potentially leading to a half-metallic ferromagnetic ground state, similar to the case of the parental compound GaSe.^56^

As the number of layers increases from single-layer to bilayer, trilayer, and ultimately to bulk, this “Mexican hat” structure gradually transforms. The associated VHS, prominent in the single-layer structure, diminishes with the addition of layers, depicted in Figure 4b,c by a reduction in the corrugation. In the bulk form of GaS, Figure 4d, the valence band dispersion transitions to a more conventional parabolic shape, and the VHS disappears. This progression indicates a significant shift in the electronic properties of GaS with increased thickness.

This transformation from a “Mexican hat” structure in monolayer GaS to a parabolic band structure in bulk GaS underscores the substantial impact of dimensional reduction on the electronic properties. This implies that GaS can be engineered to meet specific functional requirements, enhancing its potential in fields like photocatalysis, where high carrier mobility and DOS are essential for efficient performance.

**Figure 5:**
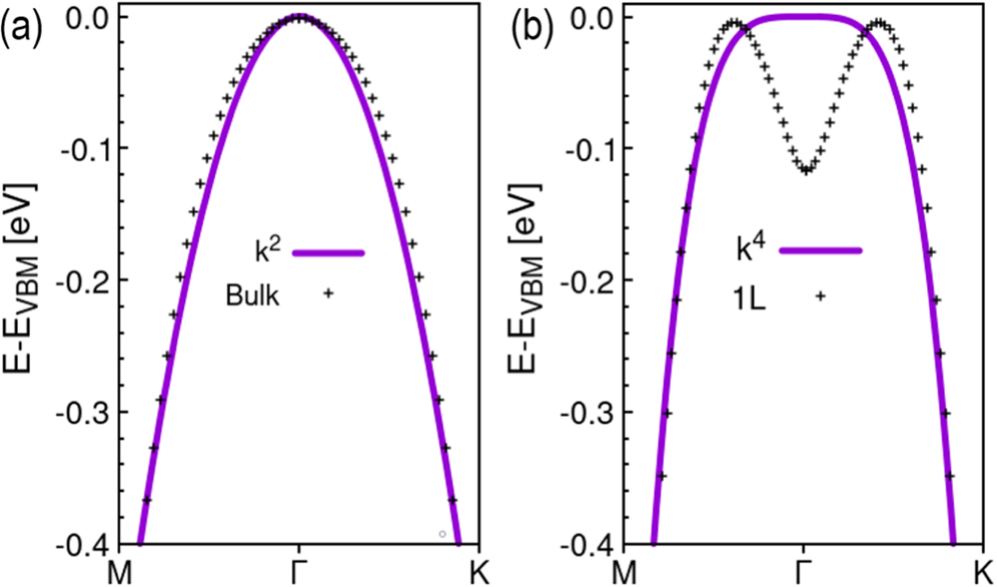
Fitting of the VBM of (a) bulk and (b) monolayer.

The theoretically predicted pudding mold-shaped energy dispersion, characterized by a quartic or higher dependence on the wave vector, has been found to exhibit better electronic and thermal properties compared to conventional parabolic dispersion. This unique shape exhibits enhanced figure of merit and power factor, making it more efficient in thermoelectric applications. The flatter regions near the valence band maximum contribute to a higher density of states, boosting electronic and thermal properties for energy conversion systems.^55^ We fitted 1L and bulk to reveal the dependence of energy on the wave vector as shown in Figure 4; the bulk shows a parabolic dependence which starts to change when the thickness is reduced to monolayer which is fitted to pudding mold band dispersion model, to wave vector of power 4 (Figure 5).

One of the main requirements for photocatalysis is that the materials must absorb a significant amount of light in the visible range. Additionally, the valence and conduction band edges of the material must align appropriately with the redox potentials of water.^57,58^ GaS satisfies these two main conditions, and several computational studies have investigated the suitability of GaS for photocatalysis in terms of band edge positions with respect to the redox potential of water.^12,59^ Specifically, under an irradiance of 0.5 mW/cm^2^, GaS-based photoelectrochemical devices exhibited a photocurrent of approximately 2.1 mA. Additionally, these devices showed a high photoresponse of 19.2 A/W, particularly notable in the UV–vis range, with the highest response observed at a wavelength of 254 nm.^7^ This optimal photocurrent and photoresponse can be traced back to the shape of the VBM and the thickness reduction, but the optimal value is only in the UV region, which represents only 5% of the sunlight spectrum. Therefore, engineering the bandgap of GaS while maintaining the intrinsic electronic structure could provide optimal absorption of visible light (>40% of the solar radiation^54,60^), which is essential in photocatalysis and solar energy applications. One strategy is to obtain a GaS monolayer with a Mexican hat-like electronic structure and a smaller effective mass, which increases the mobility; however, this Mexican hat-like band structure also increases the DOS, which enhances light absorption.

## Conclusions

Through detailed ARPES measurements we showed that the VBM is located at Γ point with isotropic dispersion below Fermi level observed by circular shape around the center of the Brillion zone. This feature changes for the deeper energy levels where the dispersion is more complex. The DFT calculations of bulk GaS further revealed that the orbital contributions of the valence bands are from Ga 4s, Ga 4p, and S 3p orbitals whereas the deeper bands are from S 3s orbitals.

We also showed by DFT calculations that the VBM in 2L, 3L and monolayer GaS exhibit a unique “pudding mold”-like dispersion, characterized by a parabolic feature. The “pudding mold” band structure predicted in 3L evolves into a “Mexican hat” configuration in the monolayer regime, which is associated with double Fermi surfaces and a higher DOS that enhances carrier mobility and a flat region at the VBM that increases the DOS. These features are critical for various applications, particularly in photocatalysis, where efficient charge transport and high DOS are paramount.

Future research should focus on experimental validation of the theoretical predictions for monolayer and few-layer GaS exploring the integration of GaS with other materials to form heterostructures with specific electronic properties.

## References

[ref1] Buscema M.; Island J. O.; Groenendijk D. J.; Blanter S. I.; Steele G. A.; van der Zant H. S. J.; Castellanos-Gomez A. Photocurrent generation with two-dimensional van der Waals semiconductors. Chem. Soc. Rev. 2015, 44 (11), 3691–3718. 10.1039/C5CS00106D. 25909688

[ref2] Younus K.; Zhou Y.; Zhu M.; Xu D.; Guo X.; Ahmed A.; Ouyang F.; Huang H.; Xiao S.; Chen Z.; He J. Observation of Anisotropic Second Harmonic Generation in Two-Dimensional Niobium Diselenide. J. Phys. Chem. Lett. 2024, 15 (18), 4992–4999. 10.1021/acs.jpclett.4c00923. 38695534

[ref3] Wang W.; Xiao Y.; Li T.; Lu X.; Xu N.; Cao Y. Piezo-photovoltaic Effect in Monolayer 2H-MoS_2_. J. Phys. Chem. Lett. 2024, 15 (13), 3549–3553. 10.1021/acs.jpclett.4c00470. 38526184

[ref4] Rodríguez Á.; Çakıroğlu O.; Li H.; Carrascoso F.; Mompean F.; Garcia-Hernandez M.; Munuera C.; Castellanos-Gomez A. Improved Strain Transfer Efficiency in Large-Area Two-Dimensional MoS_2_ Obtained by Gold-Assisted Exfoliation. J. Phys. Chem. Lett. 2024, 15 (24), 6355–6362. 10.1021/acs.jpclett.4c00855. 38857301 PMC11194808

[ref5] Lin K.; Li Y.; Ghorbani-Asl M.; Sofer Z.; Winnerl S.; Erbe A.; Krasheninnikov A. V.; Helm M.; Zhou S.; Dan Y.; Prucnal S. Probing the Band Splitting near the Γ Point in the van der Waals Magnetic Semiconductor CrSBr. J. Phys. Chem. Lett. 2024, 15 (23), 6010–6016. 10.1021/acs.jpclett.4c00968. 38814350

[ref6] Xu K.; Yin L.; Huang Y.; Shifa T. A.; Chu J.; Wang F.; Cheng R.; Wang Z.; He J. Synthesis, properties and applications of 2D layered MIIIXVI (M = Ga, In; X = S, Se, Te) materials. Nanoscale 2016, 8 (38), 16802–16818. 10.1039/C6NR05976G. 27714166

[ref7] Hu P.; Wang L.; Yoon M.; Zhang J.; Feng W.; Wang X.; Wen Z.; Idrobo J. C.; Miyamoto Y.; Geohegan D. B.; Xiao K. Highly Responsive Ultrathin GaS Nanosheet Photodetectors on Rigid and Flexible Substrates. Nano Lett. 2013, 13 (4), 1649–1654. 10.1021/nl400107k. 23465066

[ref8] Late D. J.; Liu B.; Luo J.; Yan A.; Matte H. S. S. R.; Grayson M.; Rao C. N. R.; Dravid V. P. GaS and GaSe Ultrathin Layer Transistors. Adv. Mater. 2012, 24 (26), 3549–3554. 10.1002/adma.201201361. 22678832

[ref9] Carey B. J.; Ou J. Z.; Clark R. M.; Berean K. J.; Zavabeti A.; Chesman A. S. R.; Russo S. P.; Lau D. W. M.; Xu Z.-Q.; Bao Q.; et al. Wafer-scale two-dimensional semiconductors from printed oxide skin of liquid metals. Nat. Commun. 2017, 8 (1), 14482 10.1038/ncomms14482. 28211538 PMC5336573

[ref10] Sherrell P. C.; Sharda K.; Grotta C.; Ranalli J.; Sokolikova M. S.; Pesci F. M.; Palczynski P.; Bemmer V. L.; Mattevi C. Thickness-Dependent Characterization of Chemically Exfoliated TiS2 Nanosheets. ACS Omega 2018, 3 (8), 8655–8662. 10.1021/acsomega.8b00766. 31458996 PMC6645014

[ref11] Wang B.; Gu Y.; Chen L.; Ji L.; Zhu H.; Sun Q. Gas sensing devices based on two-dimensional materials: a review. Nanotechnology 2022, 33 (25), 252001 10.1088/1361-6528/ac5df5. 35290973

[ref12] Zhuang H. L.; Hennig R. G. Single-Layer Group-III Monochalcogenide Photocatalysts for Water Splitting. Chem. Mater. 2013, 25 (15), 3232–3238. 10.1021/cm401661x.

[ref13] Harvey A.; Backes C.; Gholamvand Z.; Hanlon D.; McAteer D.; Nerl H. C.; McGuire E.; Seral-Ascaso A.; Ramasse Q. M.; McEvoy N.; et al. Preparation of Gallium Sulfide Nanosheets by Liquid Exfoliation and Their Application As Hydrogen Evolution Catalysts. Chem. Mater. 2015, 27 (9), 3483–3493. 10.1021/acs.chemmater.5b00910.

[ref14] Mosaferi M.; Sarsari I. A.; Alaei M. Band structure engineering in gallium sulfide nanostructures. Appl. Phys. A: Mater. Sci. Process. 2021, 127 (2), 123 10.1007/s00339-020-04184-z.

[ref15] Ma Y.; Dai Y.; Guo M.; Yu L.; Huang B. Tunable electronic and dielectric behavior of GaS and GaSe monolayers. Phys. Chem. Chem. Phys. 2013, 15 (19), 7098–7105. 10.1039/c3cp50233c. 23552963

[ref16] Lei S.; Wen F.; Ge L.; Najmaei S.; George A.; Gong Y.; Gao W.; Jin Z.; Li B.; Lou J.; et al. An atomically layered InSe avalanche photodetector. Nano Lett. 2015, 15 (5), 3048–3055. 10.1021/acs.nanolett.5b00016. 25822539

[ref17] Jacobs-Gedrim R. B.; Shanmugam M.; Jain N.; Durcan C. A.; Murphy M. T.; Murray T. M.; Matyi R. J.; Moore R. L.; Yu B. Extraordinary photoresponse in two-dimensional In_2_Se_3_ nanosheets. ACS Nano 2014, 8 (1), 514–521. 10.1021/nn405037s. 24359117

[ref18] Wang Y.; Szökölová K.; Nasir M. Z. M.; Sofer Z.; Pumera M. Electrochemistry of Layered Semiconducting AIIIBVI Chalcogenides: Indium Monochalcogenides (InS, InSe, InTe). ChemCatChem 2019, 11 (11), 2634–2642. 10.1002/cctc.201900449.

[ref19] Li X.; Lin M.-W.; Puretzky A. A.; Idrobo J. C.; Ma C.; Chi M.; Yoon M.; Rouleau C. M.; Kravchenko I. I.; Geohegan D. B.; Xiao K. Controlled vapor phase growth of single crystalline, two-dimensional GaSe crystals with high photoresponse. Sci. Rep. 2014, 4 (1), 5497 10.1038/srep05497. 24975226 PMC4074793

[ref20] Li X.; Basile L.; Huang B.; Ma C.; Lee J.; Vlassiouk I. V.; Puretzky A. A.; Lin M.-W.; Yoon M.; Chi M.; et al. Van der Waals epitaxial growth of two-dimensional single-crystalline GaSe domains on graphene. ACS Nano 2015, 9 (8), 8078–8088. 10.1021/acsnano.5b01943. 26202730

[ref21] Jung C. S.; Shojaei F.; Park K.; Oh J. Y.; Im H. S.; Jang D. M.; Park J.; Kang H. S. Red-to-ultraviolet emission tuning of two-dimensional gallium sulfide/selenide. ACS Nano 2015, 9 (10), 9585–9593. 10.1021/acsnano.5b04876. 26344032

[ref22] Jastrzebski C.; Olkowska K.; Jastrzebski D. J.; Wierzbicki M.; Gebicki W.; Podsiadlo S. Raman scattering studies on very thin layers of gallium sulfide (GaS) as a function of sample thickness and temperature. J. Phys.: Condens. Matter 2019, 31 (7), 075303 10.1088/1361-648X/aaf53b. 30524093

[ref23] Brenner T. M.; Egger D. A.; Kronik L.; Hodes G.; Cahen D. Hybrid organic—inorganic perovskites: low-cost semiconductors with intriguing charge-transport properties. Nat. Rev. Mater. 2016, 1 (1), 15007 10.1038/natrevmats.2015.7.

[ref24] Sze S. M.; Li Y.; Ng K. K. Physics of Semiconductor Devices; John Wiley & Sons, 2021.

[ref25] Lu H.; Chen Y.; Yang K.; Kuang Y.; Li Z.; Liu Y. Ultrafast nonlinear optical response and carrier dynamics in layered gallium sulfide (GaS) single-crystalline thin films. Front. Mater. 2021, 8, 775048 10.3389/fmats.2021.775048.

[ref26] Zappia M. I.; Bianca G.; Bellani S.; Curreli N.; Sofer Z.; Serri M.; Najafi L.; Piccinni M.; Oropesa-Nuñez R.; Marvan P.; et al. Two-Dimensional Gallium Sulfide Nanoflakes for UV-Selective Photoelectrochemical-type Photodetectors. J. Phys. Chem. C 2021, 125 (22), 11857–11866. 10.1021/acs.jpcc.1c03597. PMC8279705 34276861

[ref27] Chen H.; Li Y.; Huang L.; Li J. Intrinsic defects in gallium sulfide monolayer: a first-principles study. RSC Adv. 2015, 5 (63), 50883–50889. 10.1039/C5RA08329J.

[ref28] Demirci S.; Avazlı N.; Durgun E.; Cahangirov S. Structural and electronic properties of monolayer group III monochalcogenides. Phys. Rev. B 2017, 95 (11), 115409 10.1103/PhysRevB.95.115409.

[ref29] Whalley L. D.; Frost J. M.; Morgan B. J.; Walsh A. Impact of nonparabolic electronic band structure on the optical and transport properties of photovoltaic materials. Phys. Rev. B 2019, 99 (8), 085207 10.1103/PhysRevB.99.085207.

[ref30] Williamson B. A. D.; Buckeridge J.; Brown J.; Ansbro S.; Palgrave R. G.; Scanlon D. O. Engineering valence band dispersion for high mobility p-type semiconductors. Chem. Mater. 2017, 29 (6), 2402–2413. 10.1021/acs.chemmater.6b03306.

[ref31] Li F.; Cheng L.; Fan J.; Xiang Q. Steering the behavior of photogenerated carriers in semiconductor photocatalysts: a new insight and perspective. J. Mater. Chem. A 2021, 9 (42), 23765–23782. 10.1039/D1TA06899G.

[ref32] Kuroki K.; Arita R. Pudding mold” band drives large thermopower in NaxCoO2. J. Phys. Soc. Jpn. 2007, 76 (8), 083707 10.1143/JPSJ.76.083707.

[ref33] Kresse G.; Furthmüller J. Efficient iterative schemes for ab initio total-energy calculations using a plane-wave basis set. Phys. Rev. B 1996, 54 (16), 11169 10.1103/PhysRevB.54.11169. 9984901

[ref34] Perdew J. P.; Burke K.; Ernzerhof M. Generalized gradient approximation made simple. Phys. Rev. Lett. 1996, 77 (18), 3865 10.1103/PhysRevLett.77.3865. 10062328

[ref35] Grimme S.; Antony J.; Ehrlich S.; Krieg H. A consistent and accurate ab initio parametrization of density functional dispersion correction (DFT-D) for the 94 elements H-Pu. J. Chem. Phys. 2010, 132 (15), 154104 10.1063/1.3382344. 20423165

[ref36] Marzari N.; Mostofi A. A.; Yates J. R.; Souza I.; Vanderbilt D. Maximally localized Wannier functions: Theory and applications. Rev. Mod. Phys. 2012, 84 (4), 1419 10.1103/RevModPhys.84.1419.

[ref37] Wu Q.; Zhang S.; Song H.-F.; Troyer M.; Soluyanov A. A. WannierTools: An open-source software package for novel topological materials. Comput. Phys. Commun. 2018, 224, 405–416. 10.1016/j.cpc.2017.09.033.

[ref38] Szlachetko J.; Szade J.; Beyer E.; Błachucki W.; Ciochoń P.; Dumas P.; Freindl K.; Gazdowicz G.; Glatt S.; Guła K.; et al. SOLARIS national synchrotron radiation centre in Krakow, Poland. Eur. Phys. J. Plus 2023, 138 (1), 1–10. 10.1140/epjp/s13360-022-03592-9.

[ref39] Jie W.; Chen X.; Li D.; Xie L.; Hui Y. Y.; Lau S. P.; Cui X.; Hao J. Layer-dependent nonlinear optical properties and stability of non-centrosymmetric modification in few-layer GaSe sheets. Angew. Chem., Int. Ed. 2015, 54 (4), 1185–1189. 10.1002/anie.201409837. 25469912

[ref40] Kuhn A.; Chevy A.; Chevalier R. Crystal structure and interatomic distances in GaSe. Phys. Status Solidi A 1975, 31 (2), 469–475. 10.1002/pssa.2210310216.

[ref41] Terhell J.; Brabers V.; Van Egmond G. Polytype phase transition in the series GaSe1– xSx. J. Solid State Chem. 1982, 41 (1), 97–103. 10.1016/0022-4596(82)90039-1.

[ref42] Hahn H.; Frank G. Über die Kristallstruktur des GaS. Z. Anorg. Allg. Chem. 1955, 278 (5–6), 340–348. 10.1002/zaac.19552780516.

[ref43] Micocci G.; Rella R.; Siciliano P.; Tepore A. Investigation of electronic properties of gallium sulfide single crystals grown by iodine chemical transport. J. Appl. Phys. 1990, 68 (1), 138–142. 10.1063/1.347105.

[ref44] Borisenko E.; Borisenko D.; Bdikin I.; Timonina A.; Singh B.; Kolesnikov N. Mechanical characteristics of gallium sulfide crystals measured using micro- and nanoindentation. Mater. Sci. Eng., A 2019, 757, 101–106. 10.1016/j.msea.2019.04.095.

[ref45] Cai H.; Gu Y.; Lin Y.-C.; Yu Y.; Geohegan D. B.; Xiao K. Synthesis and emerging properties of 2D layered III–VI metal chalcogenides. Appl. Phys. Rev. 2019, 6 (4), 041312 10.1063/1.5123487.

[ref46] Barron A. R. MOCVD of group III chalcogenides. Adv. Mater. Opt. Electron. 1995, 5 (5), 245–258. 10.1002/amo.860050502.

[ref47] Brebner J. The optical absorption edge in layer structures. J. Phys. Chem. Solids 1964, 25 (12), 1427–1433. 10.1016/0022-3697(64)90057-5.

[ref48] Aulich E.; Brebner J. L.; Mooser E. Indirect Energy Gap in GaSe and GaS. Phys. Status Solidi B 1969, 31 (1), 129–131. 10.1002/pssb.19690310115.

[ref49] Terhell J. C. J. M.; Lieth R. M. A. Preparation and crystallography of gallium sulfide–selenide solid solutions. Phys. Status Solidi A 1971, 5 (3), 719–724. 10.1002/pssa.2210050327.

[ref50] Arancia G.; Grandolfo M.; Manfredotti C.; Rizzo A. Electron diffraction study of melt- and vapour-grown GaSe_1–x_S_x_ single crystals. Phys. Status Solidi A 1976, 33 (2), 563–571. 10.1002/pssa.2210330215.

[ref51] Kuhn A.; Bourdon A.; Rigoult J.; Rimsky A. Charge-density analysis of GaS. Phys. Rev. B 1982, 25 (6), 4081–4088. 10.1103/PhysRevB.25.4081.

[ref52] Kuhn A.; Chevy A.; Chevalier R. Refinement of the 2H GaS β-type. Acta Crystallogr., Sect. B: Struct. Crystallogr. Cryst. Chem. 1976, 32 (3), 983–984. 10.1107/S0567740876004445.

[ref53] De Blasi C.; Manno D.; Rizzo A. Convergent-beam electron diffraction study of melt-and vapour-grown single crystals of gallium chalcogenides. Il Nuovo Cimento D 1989, 11 (8), 1145–1163. 10.1007/BF02459022.

[ref54] Morales-García Á.; Valero R.; Illas F. An empirical, yet practical way to predict the band gap in solids by using density functional band structure calculations. J. Phys. Chem. C 2017, 121 (34), 18862–18866. 10.1021/acs.jpcc.7b07421.

[ref55] Adhidewata J. M.; Nugraha A. R.; Hasdeo E. H.; Estellé P.; Gunara B. E. Thermoelectric properties of semiconducting materials with parabolic and pudding-mold band structures. Mater. Today Commun. 2022, 31, 103737 10.1016/j.mtcomm.2022.103737.

[ref56] Cao T.; Li Z.; Louie S. G. Tunable Magnetism and Half-Metallicity in Hole-Doped Monolayer GaSe. Phys. Rev. Lett. 2015, 114 (23), 236602 10.1103/PhysRevLett.114.236602. 26196815

[ref57] Singh A. K.; Mathew K.; Zhuang H. L.; Hennig R. G. Computational screening of 2D materials for photocatalysis. J. Phys. Chem. Lett. 2015, 6 (6), 1087–1098. 10.1021/jz502646d. 26262874

[ref58] Ni M.; Leung M. K.; Leung D. Y.; Sumathy K. A review and recent developments in photocatalytic water-splitting using TiO2 for hydrogen production. Renewable Sustainable Energy Rev. 2007, 11 (3), 401–425. 10.1016/j.rser.2005.01.009.

[ref59] Cui Y.; Peng L.; Sun L.; Qian Q.; Huang Y. Two-dimensional few-layer group-III metal monochalcogenides as effective photocatalysts for overall water splitting in the visible range. J. Mater. Chem. A 2018, 6 (45), 22768–22777. 10.1039/C8TA08103D.

[ref60] Moan J. Visible light and UV radiation. In Radiation at Home, Outdoors and in the Workplace; Scandinavian Science Publisher Oslo, 2001; Vol. 2001, pp 69–85.

